# Continuous support during labour in childbirth: a Cross-Sectional study in a university teaching hospital in Shanghai, China

**DOI:** 10.1186/s12884-018-2119-0

**Published:** 2018-12-06

**Authors:** Man Wang, Qing Song, Jun Xu, Zheng Hu, Yingying Gong, Arier C. Lee, Qi Chen

**Affiliations:** 1 Department of Obstetrics & Gynaecology, Minhang District Central Hospital, Fudan University, 170 Xinsong Rd, XinZhuang, Shanghai, China; 2 Section of Epidemiology and Biostatistics, School of Population Health, The University of Auckland, 85 Park Road, Auckland, Grafton New Zealand; 3 The Hospital of Obstetrics & Gynaecology, Fudan University, Shanghai, China; 4 Department of Obstetrics & Gynaecology, The University of Auckland, 85 Park Road, Auckland, New Zealand

**Keywords:** Continuous support, Length of labour, Maternal outcomes, Chinese pregnant women

## Abstract

**Background:**

Fear or anxiety could result in adverse consequences on the course of labour. To date, family members are still not permitted in the delivery rooms in the majority of hospitals in China, and continuous support from hospital professional staff is also limited. This study aimed to evaluate the benefits of continuous support by family members and hospital professional staff during labour in China.

**Methods:**

In this Cross-Sectional study, 362 primiparous pregnancies who self-requested to receive continuous or one to one support with vaginal delivery and 362 primiparous pregnant women with routine hospital maternal care were included from a university teaching hospital. Data on the length of labour, postpartum haemorrhage (PPH), use of pain relief, use of oxytocin, fetal distress, emergency caesarean section and apgar score at 1 and 5 min were retrospectively collected from hospital medical data-base and compared between the two groups.

**Results:**

Multiple linear regressions adjusting for maternal age, BMI and birth weight, revealed the estimated length of labour for women with routine hospital maternal care was 2.03 times (95%CI 1.86 to 2.21) the duration of women with supportive care (median time, 3.05 h vs 1.5 h). In addition, Fisher’s exact test showed the emergency caesarean section rate was significantly lower in women with supportive care compared to women with routine hospital maternal care (3.3% vs 24%).

**Conclusion:**

Our results suggest that continuous support from family members together with hospital professional staff should be considered as part of intrapartum care in hospitals in China.

## Introduction

Childbirth is a life-changing experience for women, in particular for primiparous pregnant women. However, fear and anxiety are common problems faced by women during childbirth that could affect the course of labour. Studies indicate that 5–40% of pregnant women fear childbirth in Western countries [1, 2], and a recent study reported that Chinese pregnant women have moderate levels of childbirth fear and anxiety [3].

A previous study has also suggested that a home-like birth environment has beneficial effects on labour and delivery due to the presence of accompanying family members [4]. For the majority pregnant women, the hospital birthing room is a relatively clinical and foreign environment that may stimulate fear and anxiety during labour. Therefore, a satisfying birth environment can minimize maternal stress and anxiety during labour and delivery and support physiologic birth [5]. The birthing environment has been changing in recent years worldwide, including in China. However, recent studies reported that the caesarean section rate has significantly increased to 35–50% (regionally dependent) in China and the main reason for this trend was increased maternal request [6, 7]. This could be at least partially due to fear of childbirth by pregnant Chinese women.

Having family members and/or hospital professionals present continuously or one to one support the pregnant women during labour is very important, in particular for primiparous pregnant women [4, 8], and this is also recommended by the WHO (WHO recommendations: intrapartum care for a positive childbirth experience 2018). In addition to the benefits of reducing anxiety and stress, women who have continuous or one to one support from family members and/or hospital professionals such as nurses during labour and birth have better outcomes such as lower rates of caesarean section, in particular emergency caesarean section and instrumental birth, less use of pharmacological pain relief, and greater likelihood of being satisfied with their birthing experience [4, 8]. Pregnant women with continuous or one to one support during labour also have shortened labour periods, and their babies are less likely to have a low Apgar score (less than 7) at 5 min [4, 8].

Traditionally and historically in China, women have been supported by other women including female family members during childbirth. However, in last several decades in the hospital birthing environment in China, due to limited resources of health care facilities, continuous or one to one support has become difficult and is the exception rather than the routine maternal care. Continuous or one to one support from professional staff, such as midwives, to women during labour is currently not routine practice in China. Although pregnant women are currently allowed to have a companion(s) during labour in China, in most situations women are still not often allowed to bring a companion (family member) into the delivery room. A recent study found that Chinese women have high expectations of the birth environment and birth support during labour [9]. Today only few university hospitals are starting to explore continuous support during labour in few large cities in China. In addition, the outcomes of continuous support may vary by ethnicity due to culturally specific needs (Listening to Mothers III survey and [10]). To date, data investigating the beneficial effects of continuous support on women living in mainland China is limited. In this study, we performed a Cross-Sectional study to investigate the beneficial effects of continuous or one to one support during labour on primiparous pregnant women in a university teaching hospital.

## Methods

### Study setting

This Cross-Sectional study was performed in the Department of Obstetrics & Gynaecology of Minhang District Hospital, Fudan University, Shanghai, China from January 2016 to December 2016. This study was approved by the ethics committee of Minhang District Hospital. Minhang District Hospital is a university teaching hospital and during the study period, 3750 babies were born at this hospital. Minhang District Hospital began to have supportive care during labour from 2014.

### Study design and participants

This observational study was designed as a Cross-Sectional study using retrospective data from January 2016 to December 2016 from our hospital medical electronic data-base. The primary outcomes of this study were to compare the parameters of the length of labour, postpartum haemorrhage (PPH), number of use of pain relief or oxytocin, number of women had fetal distress, emergency caesarean section and Apgar score at 1 and 5 min of infants between women who received continuous support during labour and women who did not.

After excluding preterm birth (before 37 weeks of gestation) and the estimated fetal weight less than 2500 g or greater than 4200 g, 362 primiparous pregnant women who self-requested to receive continuous or one to one support during labour in our hospital were included into this study. All these participants were between 18 and 42 years old with a live singleton fetus by vaginal delivery and with more than 9 years of education, and delivered between 37 and 41 week of gestation. During the same period, after excluding women with a family history of pregnancy complications, women with multiple pregnancies, with any fetal anomaly and with risk factors in the current pregnancy as well as women with cervical dilation of over 4 cm on admission, there were 1039 health primiparous pregnant women who did not request to have continuous support before vaginal delivery. All these women had no any indications for caesarean section and had the same health conditions as women with supportive care. To match the number of women with supportive care (1,1), 362 health primiparous pregnant women who did not request to have continuous support before vaginal delivery were randomly selected from data base. Demographic data of the pregnant women in this study are summarised in Table 1.

**Table 1:** Demographic data of study population

	Study group	Control group	*P* value
Maternal age (years, median, range)	27 (18–42)	27 (17–27)	*P* = 0.614
Gestation weeks (median, range)	39^+ 6^(37–41^+ 6^)	39^+ 5^ (37–41^+ 3^)	*P* = 0.999
Birth weight (g, median, range)	3350 (2570–4360)	3340 (1920–4520)	*P* = 0.934
BMI (kg/m2) (median, range)	25.9 (17.5–36.1)	26.4 (18.6–35.6)	*P* = 0.096
Education (diploma or above) (number, %)^a^	208 (57.5%)	189 (52%)	*P* = 0.178

^a^Chi-square test was performed for the statistically analysis of education level between two group. The difference in maternal age, gestational age, birth weight and BMI between two groups was performed with Mann-Whitney U-test

The definition of continuous support is that pregnant women receive support from a family member(s) and hospital professional staff such as midwife during the labour from cervical dilation of 3 cm until 2 h after delivery and this service is one to one. The definition of routine/standard maternal care is that one professional staff member needs to support a number of pregnant women over a certain time period.

Data on the length of labour, postpartum haemorrhage (PPH), use of pain relief (yes or no), use of oxytocin (yes or no), number of women whose babies had fetal distress, emergency caesarean section and Apgar score at 1 and 5 min in study participants were collected from the hospital medical electronic data-base by two obstetricians (Wang M and Gong Y) and one midwife (Song Q) from January 2016 to December 2016 and analysed. The length of labour was calculated from cervical dilation between 3 and 4 cm with appropriate contractions, and PPH was defined as more than 500 ml of blood within the first 24 h following childbirth. PPH was calculated by the volume of blood loss during the labour and weighing the blood loss in the pad and other related materials within 24 h after birth then dividing by 1.05.

### Intervention

Followed by the hospital guideline, all women who requested continuous support from family members (mother or sisters were the majority of companions chosen, followed by the husband) and hospital professional staff (such as experienced midwives or nurses) were monitored from having appropriate contractions until 2 h after delivery. In the delivery room, family members and hospital professional staff were affectionate and provided support such as physical proximity and touch, to keep pregnant woman calm and encourage pregnant woman throughout labour and delivery. On the other hand, the control group only received the hospital’s routine/standard maternal care.

### Power of sample size

Based on the literature [11], if the estimated difference in the length of labour between intervention group and control group was 2 h ± 2.5 h (Standard deviation), 360 pregnant women in each group will provide at least 90% power with a two sided type I error rate of 5%. This was calculated by PS Power and Sample Size Calculations (Version 3.0) (http://biostat.mc.vanderbilt.edu/PowerSampleSize).

### Statistical analysis

The statistical difference in maternal age, weeks of gestation, birth weight, and BMI between two groups was assessed with a Mann-Whitney U-test using the Prism software package. The statistical difference in postpartum haemorrhage, using pain relief or oxytocin, emergency caesarean section and education level between two groups was assessed with Fisher’s exact test (or Chi-square test) using the Prism software package. The effect of supportive labour on the log transformed length of labouring adjusting for maternal age, maternal BMI, and birth-weight was analysed with multiple linear regression using SAS software, version 9.4 (SAS Institute Inc., Cary, NC, USA). Two sided *p*-values of < 0.05 were considered statistically significant.

## Results

This Cross-Sectional study was performed in the Department of Obstetrics & Gynaecology of Minhang District Hospital, Fudan University, Shanghai, China from January 2016 to December 2016. A total of 724 pregnant women participated in this study. Demographic data of the study population are summarized in Table 1. The median maternal age in the study group was 27 years (range 18–42 years). There was no statistical difference in maternal age, gestational age at delivery, BMI or education levels between the two groups. There was also no statistical difference in birth weight between the two groups (3330 g vs 3340 g, Table 1, *p* = 0.934), nor in Apgar score < 7 in the fetus at 1 (0.5% versus 0.25%, *p* = 0.999) or 5 min (0.25% versus 0%, *p* = 0.999) between the two groups.

Twelve (12/362, 3.3%) pregnant women with supportive care had an emergency caesarean section, and this rate was significantly lower than pregnant women who had routine hospital/standard maternal care (86/362, 24%) (Table 2, *p* = 0.0001). 18 (4.9%) pregnant women with supportive care used oxytocin during labour, and this was not significantly different to women who received routine hospital care (20 out of 362 cases, 5.5%) (Table 2, *p* = 0.999). 13 (3.5%) pregnant women with supportive care used pain relief during labour, but this was not significantly different to that of women who received routine hospital care (12 out of 362 cases, 3.3%) (Table 2, p = 0.999). 18 (5%) pregnant women with routine hospital care had postpartum haemorrhage, while only 8 (2.2%) pregnant women with supportive care had postpartum haemorrhage (Table 2). However, levels of PPH were not significantly different between the two groups (*p* = 0.07). 25 (7%) pregnant women with supportive care had fetal distress, while 36 (10%) pregnant women with hospital routine care had fetal distress. However there was also no statistical difference in the number of women with fetal distress between two groups (Table 2, *p* = 0.181).

**Table 2:** Summary statistics on the outcomes of labour and delivery by intervention groups

	Study group (*n* = 362)	Control group (*n* = 362)	*P* value
Length of labour (hours, median, range)	1.5 (0.16–6.1)	3.05 (0.4–11.7)	0.0001
Postpartum haemorrhage (over 500 ml) (number, %)	8 (2.5%)	18 (5%)	0.07
Use of oxytocin (number, %)	18 (4.9%)	20 (8.8%)	0.999
Use of pain relief (number, %)	13 (3.6%)	12 (3.3%)	0.999
Apgar score < 7 at 1 min (number, %)	2 (0.5%)	1 (0.25%)	0.999
Apgar score < 7 at 5 min (number, %)	1 (0.25%)	0 (0%)	0.999
Emergency caesarean section (number, %)	12 (3.3%)	86 (24%)	0.0001
Fetal distress (number, %)	25 (7%)	36 (10%)	0.181

The difference in the length of labour between two groups was performed with Mann-Whitney U-test. The differences in the proportion of PPH, use of pain relief, use of oxytocin, apagr score less than 7 at 5 min and emergence caesarean section were performed with Chi-square test

**Fig. 1:**
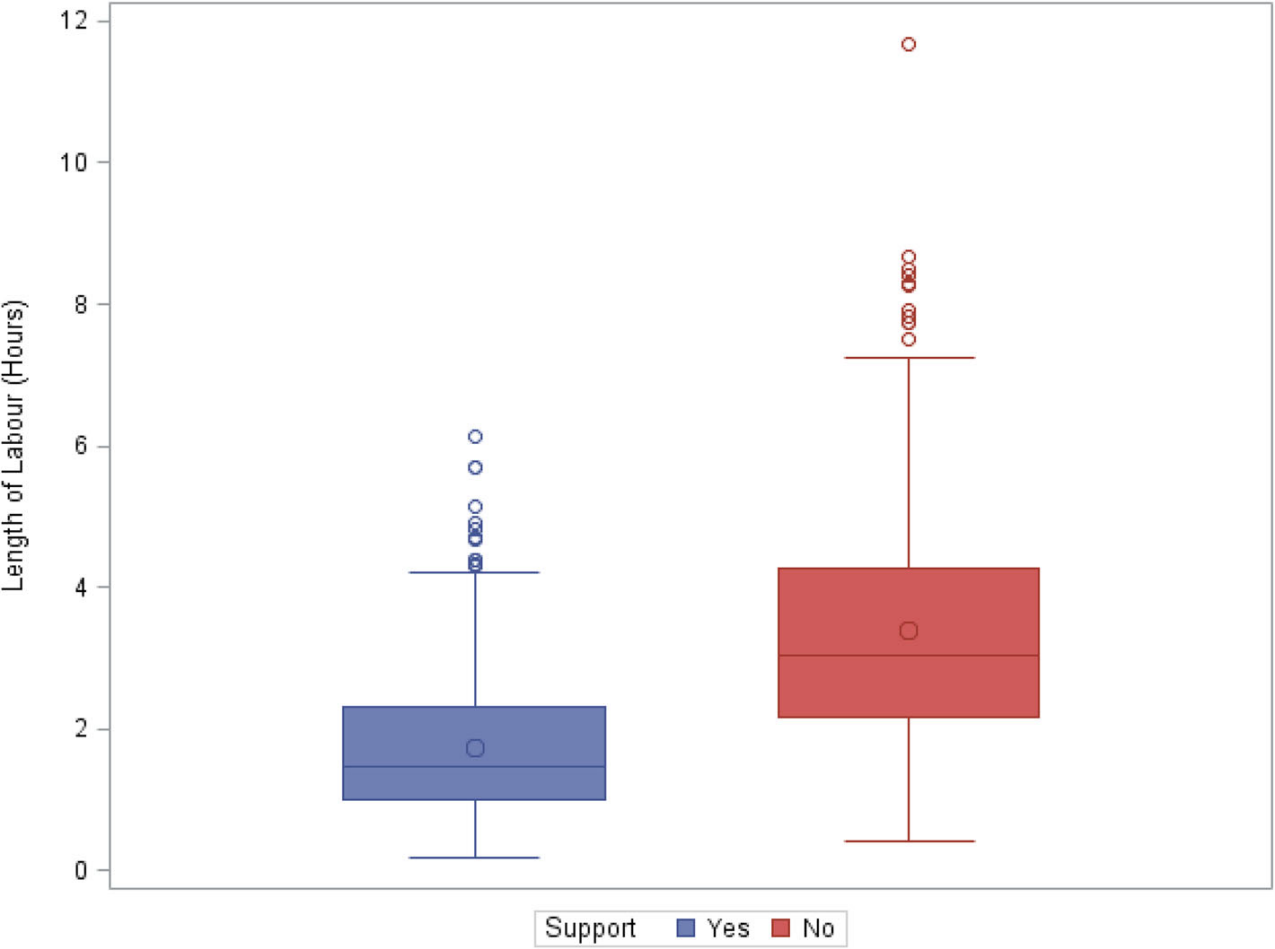
Distribution of the observed length of labour by supportive care

A Mann-Whitney U-test showed that the length of labour in women with hospital routine care was significantly longer with a median of 3.05 h (range 25 min to 11.7 h) than that in women with supportive care with a median of 1.5 h (range 10 min to 6.1 h) (Fig. 1, *p* < 0.0001). Multiple linear regression analysis was used to assess the effect of supportive care in the length of labour/delivery adjusting for maternal age, BMI and birth weight (Table 3). Due to skewness of the distribution length of labour, data was log transformed before analysis. The resulted parameter estimates and the corresponding 95% CIs were exponentially back transformed and interpreted in multiplicative terms. There was no significant association between BMI and the length of labour, however, there was a highly significant association between birth-weight and age and the length of labour (Table 3). After adjusting for maternal age, BMI and birth weight, we found that supportive labour is significantly associated with a reduction in length of labour compared to routine hospital care (Table 3, *p* < 0.0001). The estimated duration of labour for women with routine hospital care was 2.03 times (95%CI 1.86 to 2.21) the duration of the supportive labour group (Table 3, *p* < 0.0001). In addition, each 100 g increase in birthweight was associated with a 2.56% (95%CI: 1.32 to 3.81%) increase in the duration of labour. Each 10 years increase in maternal age was associated with an 18.11% (95%CI: 5.51 to 32.21%) increase in the duration of labour.

**Table 3:** Multiple linear regression result on the effect of supportive labour on length of labour, adjusting for maternal age, BMI and birth weight

Parameter	Exp (Parm. Est.)^a^	Exp (95% CI)		F Value	Pr > F
Treatment No (ref = Yes)	2.0285	1.8628	2.2091	265.29	<.0001
BMI	1.0125	0.9968	1.0286	2.42	0.1199
Birth weight each100g increase	1.0256	1.0132	1.0381	16.61	<.0001
Maternal age each 10 years increase	1.1811	1.0551	1.3221	8.39	0.0039

^a^Due to skewness of the distribution length of labour was log transformed before analysed using multiple linear regressions. The resulted parameter estimates and 95% CI of the parameter estimates were back transformed using exponential function

## Discussion

In our current study with large sample size (*n* = 724) after adjusting for maternal age, BMI and birth weight, our data shows that the length of labour/delivery (cervical dilation from 3 cm until birth) was significantly shorter in primiparous pregnant women with the support of both a family member and hospital professional staff support, compared to primiparous pregnant women with routine maternal care (1.5 h versus 3.05 h). A study with small sample size (*n* = 50) led by Kashanian showed that there was a shorter duration of first and second stage of labour, but not third stage of labour, in women with continuous or one to one support [12]. Another study led by Khresheh showed that there was a shorter duration in the whole labour period for women with continuous or one to one support but it did not reach statistical significance [11], potentially due to the smaller sample size (*n* = 107). In addition, the variation in the different studies could be due to the definition of the supportive companion being a female relative without medical or nurse experience in Khresheh’s study, while in Kashanian’s study, the supportive companion was a professional midwife. In our current study with 362 participants, the supportive companions included both family member(s) and a professional midwife. There may be a difference between the support from a professional staff such as midwife and the support from a family member or a friend during labour [13]. Studies suggested that women with a partner present during labour were less likely to fear childbirth [14] and women with a partner present during labour had increase their satisfaction with the experience [8, 13]. In addition, a recent study also suggested that women preferred someone with whom they were familiar and comfortable [15]. However, our hospital guideline in continuous support during labour indicates that professional staff must be with pregnant women throughout labour and delivery. Therefore in our current study we were not able to analyse the difference in the outcomes of labour between women who received the support from professional staff and women who only received the support from a family member(s). However, whether there is a difference in the outcomes of labour made by the different supportive companions needs to be further investigated in future. In our current study we monitored the length of birth from 3 cm of cervical dilation until birth, which suggests our study covered both the first and second stages of labour and our study supports Khresheh’s study.

Although in our current study we found that there was a highly significant association between birth-weight and the length of labour, as well as the association between age and the length of labour, there was no difference in the birth-weight and age between two groups. Pregnant women with continuous or one to one support are likely to have less stress or fear or anxiety during labour, consequently these women are likely less to use oxytocin [16]. In Kashanian’s study, the proportion of women using oxytocin was higher in pregnant women without continuous or one to one support [12]. However, in our current study, our data showed that the number of pregnant women who used pain relief or use of oxytocin during labour and delivery was not different between women with continuous or one to one support and pregnant women with routine hospital care. This could be because there is currently an education program on the labour/delivery process in our hospital which could reduce the fear or anxiety of primiparous pregnant women. Taken together, our data further suggests that continuous or one to one support may be a main reason for reducing labour time.

Today women are more concerned about labour than ever before and this fear is related to several serious conditions such as prolonged labour, a greater need for pain relief during labour and an increased risk of an emergency caesarean section [17]. Studies found that women who suffer from fear of childbirth during pregnancy have an increased rate of emergency caesarean sections or more complicated vaginal deliveries involving vacuums or other instruments [18, 19]. It is well documented that longer labour/delivery may lead to an emergency caesarean section [19] and that women with low fear of childbirth are likely to have vaginal delivery with the most positive birth experience [17]. In our current study, we also found that only 12 (3.3%) women with supportive care required an emergency caesarean section, while 86 (24%) women with routine hospital care required an emergency caesarean section which was significantly higher. In combination with other studies, our data suggests that supportive care during labour can empower women by reducing their fears.

Due to the Chinese traditional culture, the pregnant woman’s mother or sister is usually chosen as the companion in this study. This may result in the variations in the results when compared to other studies as it has previously been shown that support from the husband or male partner is positively associated with the labour/delivery process and significantly reduce the likelihood caesarean section [20]. Further study is required in Chinese women.

Continuous or one to one support from family members and/or hospital professional staff can give a pregnant woman a continuous monitoring during labour. Fetal distress is estimated to occur in 1–4% of pregnant women during labour and a quick labour usually is the way to relieve fetal distress by putting excessive stress on the fetus [21–23]. In our current study, our data shows that there was a 7% fetal distress rate in women who received supportive care which was slightly lower, but not significantly than, women with routine hospital maternal care (10%). Using oxytocin can over-stimulate the uterus resulting in distress of the fetus [24], but there was no difference in using oxytocin during labour/delivery between two groups in our study suggesting quicker labour in women with supportive care could be one of the reasons for this slightly lower fetal distress.

We acknowledge that there are some limitations of the study. This is a Cross-Sectional study analysing retrospective data, not a randomised study, the control group (362 women with hospital routine care) was randomly selected from 1039 health primiparous pregnant women who had hospital routine care. In addition, 362 health primiparous pregnant women in study group self-requested to receive continuous supportive care. This may cause a selection bias.

## Conclusion

Education on the labour/delivery process is starting to be routinely provided at admission in most hospitals in China, but does not occur in all regions. To date the family members are still not permitted in the delivery rooms in the majority of hospitals in China due to the limitation of maternal care facilities and Chinese culture. In conclusion, our results demonstrate that there is a significantly reduction in the length of labour and emergency caesarean section rate in women with continuous support from family member and professional staff. However, this clinical practice does not reduce other complications during the labour. Future prospective studies are required to confirm our findings. Our results suggest that this clinical practice should be considered as part of intrapartum care in hospitals in China and other developing countries where such services are currently not available.

## Abbreviations

PPH: Postpartum haemorrhage

## References

[1] Nieminen K, Stephansson O, Ryding EL (2009). Women’s fear of childbirth and preference for cesarean section--a cross-sectional study at various stages of pregnancy in Sweden. Acta Obstet Gynecol Scand.

[2] Kringeland T, Daltveit AK, Moller A (2009). What characterizes women in Norway who wish to have a caesarean section?. Scand J Public Health.

[3] Gao LL, Liu XJ, Fu BL, Xie W (2015). Predictors of childbirth fear among pregnant Chinese women: a cross-sectional questionnaire survey. Midwifery.

[4] Hodnett ED, Gates S, Hofmeyr GJ, Sakala C (2013). Continuous support for women during childbirth. Cochrane Database Syst Rev.

[5] Stark MA, Remynse M, Zwelling E (2016). Importance of the birth environment to support physiologic birth. J Obstet Gynecol Neonatal Nurs : JOGNN / NAACOG.

[6] Wang X, Hellerstein S, Hou L, Zou L, Ruan Y, Zhang W (2017). Caesarean deliveries in China. BMC Pregnancy Childbirth.

[7] Li H, Luo S, Trasande L (2017). Geographic variations and temporal trends in cesarean delivery rates in China, 2008-2014. JAMA.

[8] Bohren MA, Hofmeyr GJ, Sakala C, Fukuzawa RK, Cuthbert A (2017). Continuous support for women during childbirth. Cochrane Database Syst Rev.

[9] Zhang X, Lu H (2014). Childbirth expectations and correlates at the final stage of pregnancy in Chinese expectant parents. Int J Nurs Sci.

[10] Greenberg MB, Cheng YW, Hopkins LM, Stotland NE, Bryant AS, Caughey AB (2006). Are there ethnic differences in the length of labor?. Am J Obstet Gynecol.

[11] Khresheh R (2010). Support in the first stage of labour from a female relative: the first step in improving the quality of maternity services. Midwifery.

[12] Kashanian M, Javadi F, Haghighi MM (2010). Effect of continuous support during labor on duration of labor and rate of cesarean delivery. Int J Gynaecol Obstet.

[13] Kabakian-Khasholian T, Portela A (2017). Companion of choice at birth: factors affecting implementation. BMC Pregnancy Childbirth..

[14] Hinton L, Locock L, Knight M (2014). Partner experiences of “near-miss” events in pregnancy and childbirth in the UK: a qualitative study. PLoS One.

[15] Lunda P, Minnie CS, Benade P (2018). Women’s experiences of continuous support during childbirth: a meta-synthesis. BMC Pregnancy Childbirth.

[16] Hatem M, Sandall J, Devane D, Soltani H, Gates S. Midwife-led versus other models of care for childbearing women. Cochrane Database Syst Rev. 2008;8(4):36–47. 10.1002/14651858.CD004667.pub2 18843666

[17] Elvander C, Cnattingius S, Kjerulff KH (2013). Birth experience in women with low, intermediate or high levels of fear: findings from the first baby study. Birth.

[18] Johnson R, Slade P (2002). Does fear of childbirth during pregnancy predict emergency caesarean section?. BJOG.

[19] Ryding EL, Wijma B, Wijma K, Rydhstrom H (1998). Fear of childbirth during pregnancy may increase the risk of emergency cesarean section. Acta Obstet Gynecol Scand.

[20] McGrath SK, Kennell JH (2008). A randomized controlled trial of continuous labor support for middle-class couples: effect on cesarean delivery rates. Birth.

[21] Penning S, Garite TJ (1999). Management of fetal distress. Obstet Gynecol Clin N Am.

[22] Garite TJ, Simpson KR (2011). Intrauterine resuscitation during labor. Clin Obstet Gynecol.

[23] Chauhan SP, Magann EF, Scott JR, Scardo JA, Hendrix NW, Martin JN (2003). Cesarean delivery for fetal distress: rate and risk factors. Obstet Gynecol Surv.

[24] Liston WA, Campbell AJ (1974). Dangers of oxytocin-induced labour to fetuses. Br Med J.

